# Design and Fabrication of a High-Sensitivity and Wideband Cymbal Hydrophone

**DOI:** 10.3390/s23229086

**Published:** 2023-11-10

**Authors:** Donghyun Kim, Yongrae Roh

**Affiliations:** School of Mechanical Engineering, Kyungpook National University, Daegu 41566, Republic of Korea; roy4435@naver.com

**Keywords:** cymbal hydrophone, high sensitivity, broadband characteristics, receiving voltage sensitivity (RVS), optimal design

## Abstract

So far, cymbal transducers have been developed primarily for transmitting purposes, and even when used for receiving, the focus has been mostly on improving the receiving sensitivity. In this study, we developed a cymbal hydrophone with a higher sensitivity and a wider bandwidth than other existing hydrophones. First, the initial structure of the cymbal hydrophone was established, and then the effects of structural variables on the hydrophone’s performance were analyzed using the finite element method. Based on the analysis results, the structure having the highest sensitivity and widest bandwidth, with a receiving voltage sensitivity level above a certain threshold, was derived using optimal design techniques. A prototype of the cymbal hydrophone with the designed structure was fabricated, and its performance was measured, validating the effectiveness of the design by comparing the measurement results with the design values. The developed cymbal hydrophone is expected to be utilized in various underwater precision measurements, as it possesses a significantly broader reception frequency bandwidth when compared with other hydrophones used for the same purpose.

## 1. Introduction

A cymbal transducer is a simple structure consisting of a piezoceramic disk sandwiched between two cymbal-shaped metal caps [1]. The size of the cymbal transducer is small compared to the wavelength at its resonant frequency [2]. Its acoustic properties vary depending on the dimensions and material of the metal cap, even when using the same-size piezoelectric element [3]. Due to the reversibility of the piezoelectric effect, the cymbal transducer can be utilized as both a projector and a hydrophone. However, while many studies use cymbal transducers for transmitting, there are not many studies that use them for receiving.

When the cymbal transducer is employed as a hydrophone, it operates based on a distinct mechanism. Sound pressure originating from an external source is directed towards the cymbal hydrophone, leading to the deformation of the hydrophone’s cap and the application of radial stresses to the piezoceramic disk. In response, the piezoceramic disk generates an electrical voltage through the direct piezoelectric effect. This operational principle relies on the metal cap serving as a mechanical transformer, converting and amplifying the force originally applied in the axial direction of the piezoceramic disk into a radial force. This transformation significantly contributes to the high sensitivity of the cymbal hydrophone [4]. Furthermore, the cymbal hydrophone’s caps play a crucial role in adapting the high acoustic impedance of the piezoceramic material to the low acoustic impedance of the surrounding medium. This process, known as impedance matching, has a substantial impact on the widening of the receiving frequency bandwidth of the cymbal hydrophone. Efficient impedance matching enables the seamless transfer of acoustic energy between the hydrophone and its surroundings, resulting in improved sensitivity and an expanded range of frequencies that the hydrophone can capture [5]. To use a piezoelectric transducer as a hydrophone, high sensitivity and broadband characteristics are crucial, and in this regard, the cymbal hydrophone can outperform other hydrophones.

In previous research on cymbal hydrophones, Chen et al. [4] introduced a cymbal hydrophone structure aimed at enhancing stability in reception and achieving a broader frequency bandwidth for receiving signals. Additionally, Kannan et al. [6] conducted an analysis of the changes in receiving voltage sensitivity (RVS) and bandwidth of a cymbal hydrophone in relation to its structure, and they compared these analytical findings with measurements obtained from a prototype. Evaluating the performance of cymbal hydrophones, Li et al. explored planar series and parallel arrangements of cymbal transducers for hydrophone applications [7]. In separate works, Li et al. [8] utilized PMN-0.33PT, and Lonkar et al. [9] employed PNS-PZT piezoelectric single crystals to propose cymbal hydrophones. They fabricated prototype hydrophones and compared their performance against that of a cymbal transducer made of PZT. However, it is important to note that most of the aforementioned studies focused on altering the material or structure of the transducer solely to increase the RVS of the hydrophone. As a result, there has not been a comprehensive analysis and design approach to enhance overall hydrophone characteristics, including both RVS and the receiving frequency bandwidth.

Our primary goal was to develop a cymbal hydrophone structure that could simultaneously exhibit high RVS and a wide bandwidth. To achieve this objective, we conducted an in-depth analysis of the hydrophone’s performance by employing the finite element method (FEM) to study the influence of various structural parameters. Based on the insights gained from finite element analysis (FEA), we devised a hydrophone structure that maximized both sensitivity and bandwidth while ensuring that the RVS level at a specific frequency surpassed a certain threshold and that the peak RVS frequency aligned with our desired value.

Subsequently, we proceeded to fabricate a prototype of the designed hydrophone. The performance of this prototype was carefully measured, and the obtained results were then compared with the simulation data. This comparison allowed us to validate the effectiveness and accuracy of our design approach. By successfully achieving a hydrophone with improved RVS and a wide bandwidth, our study contributes to a more comprehensive and refined understanding of cymbal hydrophone characteristics.

## 2. Structure and Model Validation of the Cymbal Hydrophone

**Figure 1:**
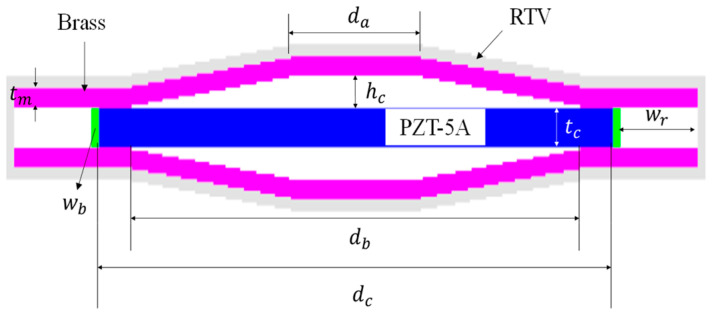
2D FEA model of the cymbal hydrophone.

In this study, a commercial program, PZFlex^®^, was used to build a finite element analysis model of a cymbal hydrophone and to analyze its acoustic properties. Figure 1 is a 2D model of the cymbal hydrophone. A hard-plastic ring was attached to the piezoceramic disk to improve the structural robustness of the hydrophone [10]. The parameters *d*_a_, *d*_b_, and *d*_c_ represent the diameters of the cavity apex, cavity base, and piezoceramic disk, respectively. The parameters *t*_m_ and *t*_c_ are the thicknesses of the metal cap and piezoceramic disk, respectively, while *h*_c_, *w*_r_, and *w*_b_ are the height of the cavity and the widths of the ring and the bond, respectively. The cymbal hydrophone had a structure in which metal caps adhered to the upper and lower surfaces of the piezoceramic disk. The adhesive layer between the metal cap and the piezoceramic disk was so thin that it was ignored in the analytical model [11]. For underwater application, the hydrophone was insulated with a polymer coating with a thickness of 0.3 mm. The materials of the piezoceramic disk, metal cap, and ring were PZT-5A, brass, and polyetheretherketone, respectively. The properties of the materials were taken from Reference [12]. Table 1 shows the dimensions of an initial hydrophone model. The initial dimensions were estimated to achieve the peak RVS frequency targeted in this study based on the results of our previous studies [13,14].

**Figure 2:**
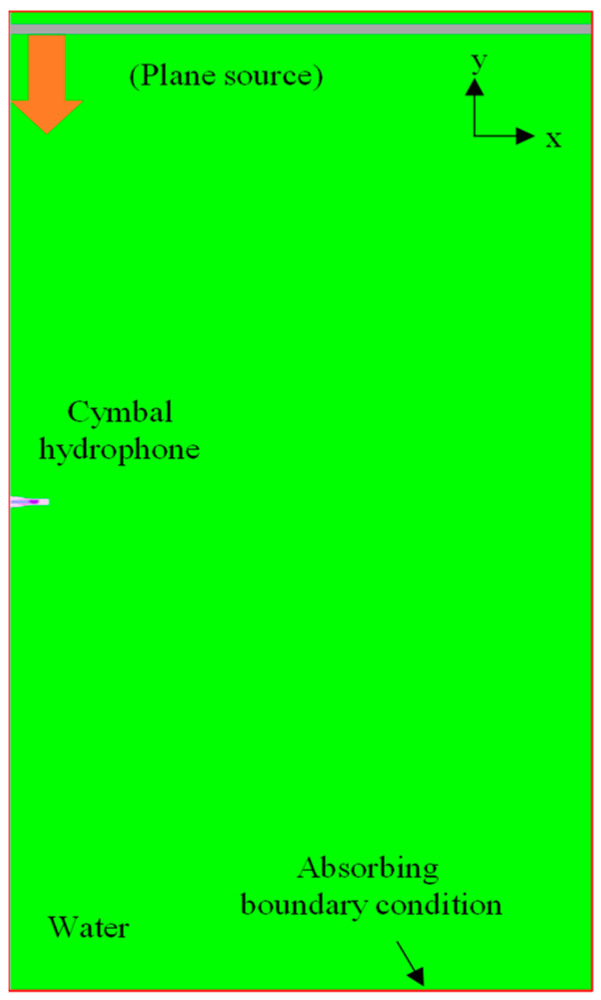
Model for the underwater performance analysis of the cymbal hydrophone.

**Figure 3:**
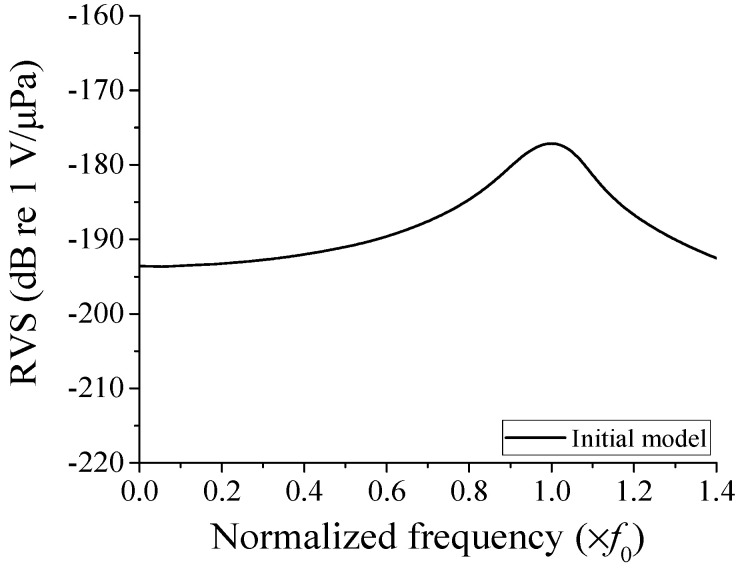
RVS spectrum of the initial cymbal hydrophone model.

First, the RVS of the cymbal hydrophone was analyzed using the initial model with the dimensions in Table 1. The output voltage generated by the hydrophone was calculated using the underwater sound wave propagation model in Figure 2. A plane wave of 1 Pa was transmitted toward the hydrophone via a plane source. A sufficient amount of water was set up to ensure far-field wave propagation from the source. An element size of 0.15 mm was used to construct the model. The entire model consisted of 5.14 million elements and 5.15 million nodes, reflecting the complexity and precision of the analysis. A sound-absorbing boundary condition was enforced on the edges of the water domain to prevent sound reflection. The sound pressure *P*_i_ applied to the cymbal hydrophone and the voltage *V*_out_ induced in the hydrophone was substituted into Equation (1) to calculate the RVS. Figure 3 is the RVS spectrum of the initial hydrophone model, where the frequency on the horizontal axis was normalized to *f*_0,_ at which the RVS level peaked. In Figure 3, the cymbal hydrophone showed very flat frequency characteristics in the low-frequency band. The performance factors extracted from the RVS spectrum were the peak RVS frequency, the RVS level at the lowest frequency in the graph, and the receiving frequency bandwidth. The peak RVS frequency of the initial hydrophone was *f*_0_, the RVS level at 0.01*f*_0_ was −193.6 dB, and the frequency bandwidth with an RVS level difference within 3 dB from that at 0.01*f*_0_ was 0.565*f*_0_.

(1) $$RVS=20\log\left(V_{out}/P_{i}\right)\left(dB\;re\;1\;V/\mu Pa\right)$$

**Figure 4:**
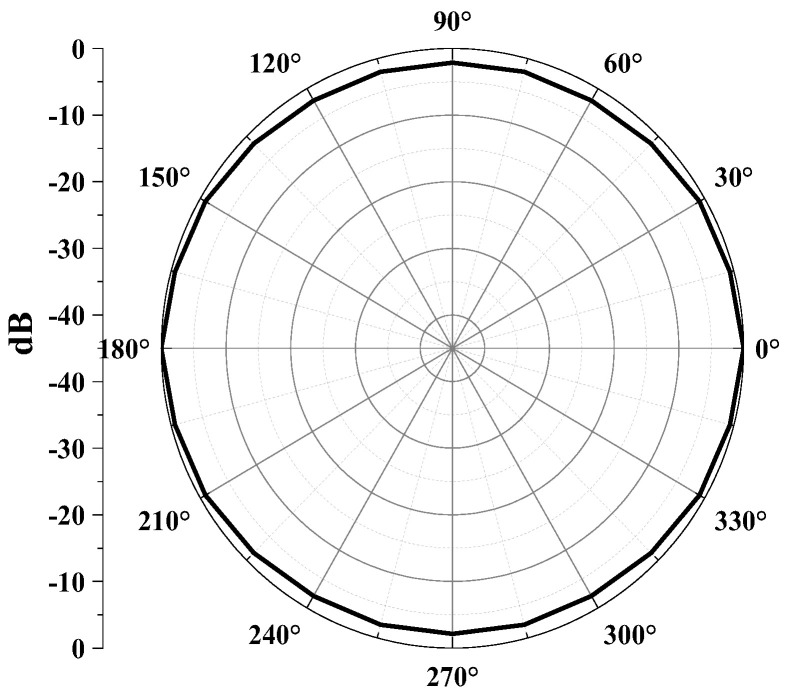
Beam pattern of the cymbal hydrophone.

Next, we examined the received beam pattern to verify the directionality of the cymbal hydrophone. In Figure 2, we systematically rotated the orientation of the plane source from 0° to 90° in 7.5° increments, maintaining a consistent distance between the hydrophone’s center and the plane source. We transmitted a tone burst signal that was centered at a frequency of 0.33*f*_0_ to the hydrophone. The beam pattern *b*(*θ*) was computed by substituting the output voltage at each direction into Equation (2), where *V*_θ_ represents the output voltage of the cymbal hydrophone at each azimuth angle *θ*, and *V*_max_ indicates the maximum value among the *V*’s for all directions. The resulting beam pattern is illustrated in Figure 4. It is worth noting that the radius of the cymbal hydrophone is very small, approximately 1/23 of the wavelength of the transmitted sound wave. Consequently, the hydrophone exhibits an omnidirectional beam pattern [15].

(2) $$b(\theta )=20\;\log_{10}\left|\frac{V_{\theta }}{V_{max}}\right|$$

## 3. Effect of the Structural Parameters on the Cymbal Hydrophone Performance

The aim of this study is to design a cymbal hydrophone with specific attributes: a desired peak RVS frequency, an RVS level exceeding a predefined threshold, and a wide bandwidth. To achieve this goal, we scrutinized the impact of structural variables on the hydrophone’s performance. In our preliminary investigation, we identified that the most influential parameters for the acoustic characteristics of cymbal transducers were *d*_a_, *d*_b_, *h*_c_, and *t*_m_ [13]. However, we had to exclude *t*_m_ from the effect analysis due to limitations in the metal cap processing tolerance, so it was set at a fixed value of 0.5 mm. To investigate the influence of these variables, *d*_a_ and *d*_b_ were varied within a range of ±10% from the dimensions of the initial model. On the other hand, *h*_c_ was adjusted within a range of ±40% from the initial dimensions, taking into account machining cap tolerances.

**Figure 5:**
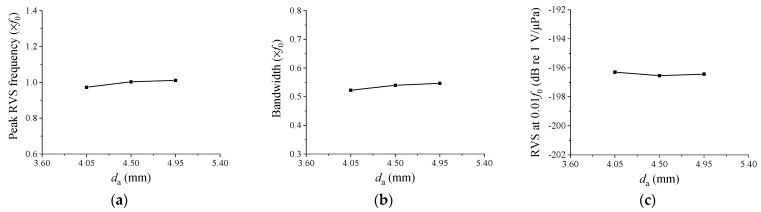
Variation of the performance in relation to *d*_a_: (**a**) peak RVS frequency, (**b**) bandwidth, (**c**) RVS at 0.01*f*_0_.

Initially, we examined the impact of changing *d*_a_ on hydrophone performance, and the results are depicted in Figure 5. As *d*_a_ increased, the peak RVS frequency and bandwidth displayed slight increases, but the variation in the RVS at 0.01*f*_0_ was negligible. In summary, *d*_a_ did not significantly affect the hydrophone’s performance, and consequently it was excluded from the variables considered for subsequent design modifications.

**Figure 6:**
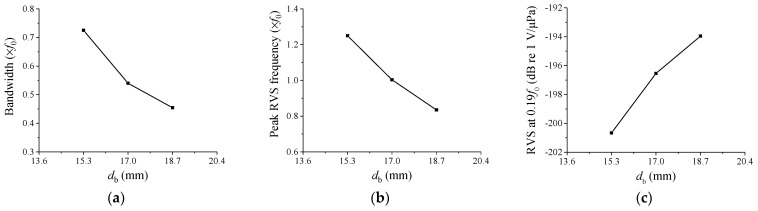
Variation of the performance in relation to *d*_b_: (**a**) peak RVS frequency, (**b**) bandwidth, (**c**) RVS at 0.01*f*_0_.

Figure 6 illustrates the alteration in acoustic characteristics of the hydrophone in response to changes in *d*_b_. The variable *d*_b_ was adjusted within the range of 15.3–18.7 mm, with intervals of 1.7 mm. As *d*_b_ increased, the peak RVS frequency and bandwidth decreased rapidly. Although the area of the piezoceramic disc remained constant, an increase in *d*_b_ resulted in a reduced attachment area for the cap along the ceramic’s edge, thereby decreasing the overall stiffness of the hydrophone. This increased the cap’s vibrations, lowering the peak RVS frequency. The reduction in the peak RVS frequency also led to a corresponding decrease in bandwidth. Moreover, the RVS level at 0.01*f*_0_ increased significantly with the increase in *d*_b_. This was primarily because a lower stiffness caused more cap vibrations, transmitting greater physical changes to the piezoceramic disk, thereby boosting sensitivity. Consequently, *d*_b_ proved to be a valuable variable as it had a substantial impact on all of the key performance metrics.

**Figure 7:**
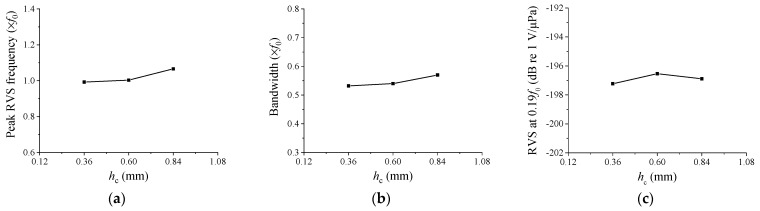
Variation of the performance in relation to *h*_c_: (**a**) peak RVS frequency, (**b**) bandwidth, (**c**) RVS at 0.01*f*_0_.

In Figure 7, we explore the impact of *h*_c_ on hydrophone performance. The initial *h*_c_ dimension was 0.60 mm, and it was adjusted from 0.36 mm to 0.84 mm in 0.24 mm increments. As *h*_c_ increased, both the peak RVS frequency and bandwidth expanded. This was due to the steeper slope of the cap’s sides as *h*_c_ increased, which also increased the effective stiffness of the cap. Higher stiffness resulted in reduced cap vibrations and a higher peak RVS frequency for the hydrophone, thus broadening the bandwidth. Although the RVS level at 0.01*f*_0_ showed a peak value at a specific *h*_c_, the difference was not substantial. Based on these findings, *h*_c_ was deemed a valuable variable for fine-tuning the performance of the cymbal hydrophone.

From the findings presented above, it is evident that the influences of these structural variables are not isolated but interconnected. Consequently, to achieve a cymbal hydrophone with both a high sensitivity and a wide bandwidth, the focus should not solely be on determining specific values for individual variables. Instead, the key lies in identifying the optimal combination of these variables. Therefore, we employed an optimization process, which is elaborated upon in the subsequent section, to determine the most advantageous combination of design variables.

## 4. Optimal Design of the Broadband Cymbal Hydrophone Structure

In the preceding section, we discovered that the effects of structural variables on hydrophone performance are not standalone but are intricately interconnected. Consequently, to identify the optimal combination of these variables while considering their interdependencies, we conducted a statistical multiple regression analysis using the data presented in Figure 5, Figure 6 and Figure 7 [16]. Based on the outcomes in Section 3, we chose *d*_b_ and *h*_c_ as the design variables for our optimization efforts. The range for optimizing *d*_b_ was set between 15.3 mm and 18.7 mm, and for *h*_c_, it was set between 0.36 mm and 0.84 mm, mirroring the ranges used in the trend analysis.

The overarching aim of this study is to design a cymbal hydrophone that possesses a specified peak RVS frequency, maintains an RVS level exceeding a certain threshold at a specific frequency, and offers the broadest achievable bandwidth. Accordingly, we formulated our objective function as per Equation (3), and we set the corresponding constraints as outlined in Equation (4). These constraints stipulate that the desired peak RVS frequency must fall within a tolerance of ±0.03*f*_0_, and the RVS level at 0.01*f*_0_ must surpass that of the initial model.

Objective function: minimize (|RVS level at 0.01*f*_0_|/bandwidth) (3)

Constraints: ① 1.17*f*_0_ ≤ peak RVS frequency ≤ 1.23*f*_0_ ② RVS level at 0.01*f*_0_ ≥ −193.3 dB (4)

For the optimization process, we employed the 3^k^ experimental design method to select and analyze a total of 9 cases [17]. We conducted multiple regression analyses on the gathered data to derive regression functions for both the objective function and the constraints [18]. Subsequently, we applied the OptQuest Nonlinear Programs (OQNLP) algorithm to determine the optimal combination of design variables that adhered to the specified objective function and constraints, as summarized in Table 2 [19].

**Figure 8:**
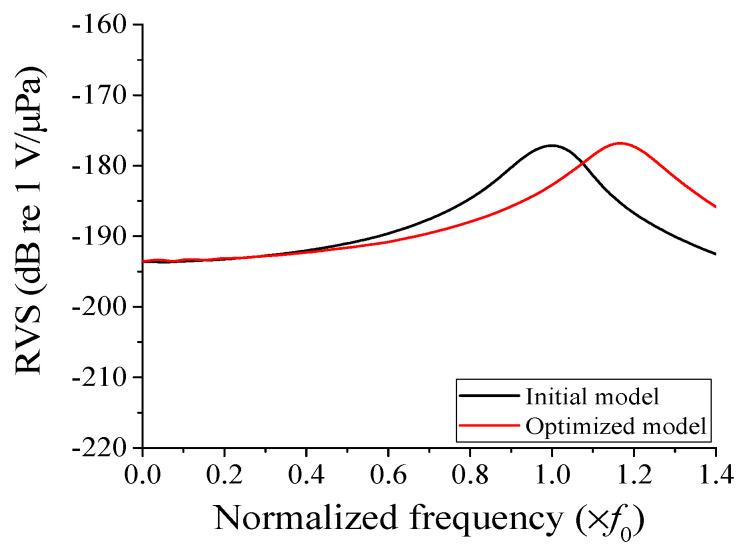
Comparison of the RVS spectra of the initial and optimized cymbal hydrophones.

Figure 8 illustrates the performance of the hydrophone with the optimized structure in comparison to the initial model, and Table 3 provides a quantitative assessment of the performance differences. The optimized model exhibited an increased bandwidth of approximately 0.1*f*_0_ compared to the initial model, along with a 0.13 dB boost in the RVS level at 0.01*f*_0_. It is worth noting that a wider bandwidth naturally accompanies an increase in peak RVS frequency. To facilitate a clear comparison, we evaluated the bandwidth relative to peak RVS frequency. In terms of the fractional bandwidth, the optimized model demonstrated an 8.8% increase compared to the initial model. Additionally, the peak RVS frequency of the optimized cymbal hydrophone reached 1.17*f*_0_, while the RVS level at 0.01*f*_0_ was −193.17 dB, satisfying all the design conditions. This confirmed the effectiveness of the design approach outlined in this study.

## 5. Fabrication and Characterization of Cymbal Hydrophone

**Figure 9:**
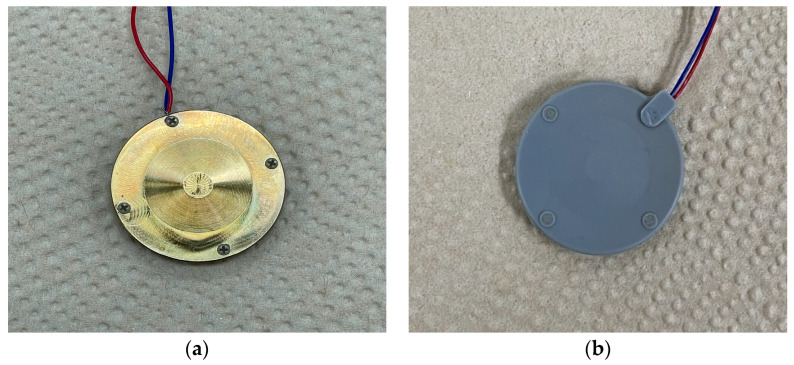
Fabricated cymbal hydrophone prototype: (**a**) before coating, (**b**) after coating.

**Figure 10:**
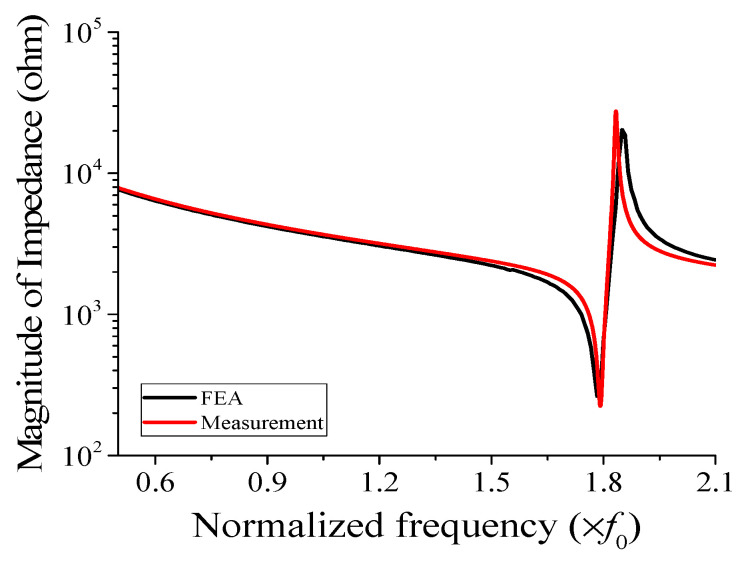
Comparison of the measured and simulated impedance spectra of the cymbal hydrophone in air.

To validate the feasibility of the structure outlined in Section 4, we constructed a prototype of the cymbal hydrophone with the designed configuration and conducted performance measurements. The prototype was fabricated to match the dimensions and materials specified earlier. Initially, the brass cap, piezoceramic, and plastic ring were assembled using epoxy (EB-106, EpoxySet, Inc., Woonsocket, RI, USA) [14]. Subsequently, the prototype was coated with RTV-3460 (Elkem, Oslo, Norway) for waterproof insulation. Figure 9 is a photograph of the coated cymbal hydrophone prototype. We then measured the impedance of the prototype in air using an impedance analyzer (Agilent 4294A, Santa Clara, CA, USA) and compared it with the spectrum that was simulated via FEM, as depicted in Figure 10. According to the FEA, the resonant and anti-resonant frequencies of the cymbal hydrophone were 1.78*f*_0_ and 1.85*f*_0_, respectively, while the measurements yielded 1.79*f*_0_ and 1.83*f*_0_, respectively. The agreement between the two spectra was strong, confirming that the cymbal hydrophone prototype was manufactured precisely in accordance with the design specifications.

**Figure 11:**
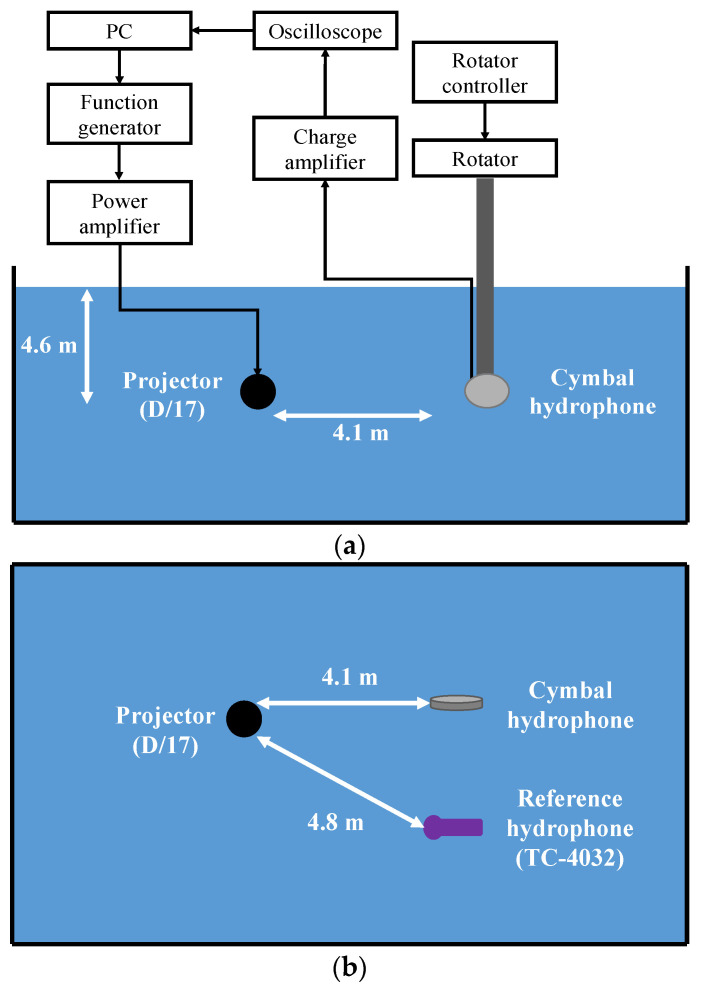
Comparison Schematic of the experiment setup: (**a**) side view, (**b**) top view.

Subsequently, we measured the RVS and received the beam pattern of the hydrophone prototype using the methodology described in [20] within the environment depicted in Figure 11. To minimize wave reflection from the walls of the water tank, we covered all of the interior surfaces of the tank with sound-absorbing material. The input signal was generated using a function generator, amplified with a power amplifier, and applied to a standard projector (D/17; Neptune Sonar, Kelk, UK). These generated waves reached both the cymbal hydrophone prototype and a standard hydrophone (TC4032; Teledyne) simultaneously [21]. The resulting output voltages from the hydrophones were recorded on a control computer and were subsequently analyzed to assess the RVS spectra and receive beam patterns. The standard projector and the cymbal hydrophone were positioned 4.1 m apart, facing each other at the same depth of 4.6 m. The standard hydrophone served multiple purposes: it measured sound pressure for accurate RVS calculations, monitored sound wave distortion and reflections, and calibrated the RVS of the cymbal hydrophone. The standard hydrophone was placed 4.8 m from the projector at a water depth of 4.6 m. For the RVS measurement, we maintained the same configuration as the FEA model and measured the RVS at 100 Hz intervals, starting from 0.27*f*_0_, which was the lowest frequency measurable within the experimental environment.

**Figure 12:**
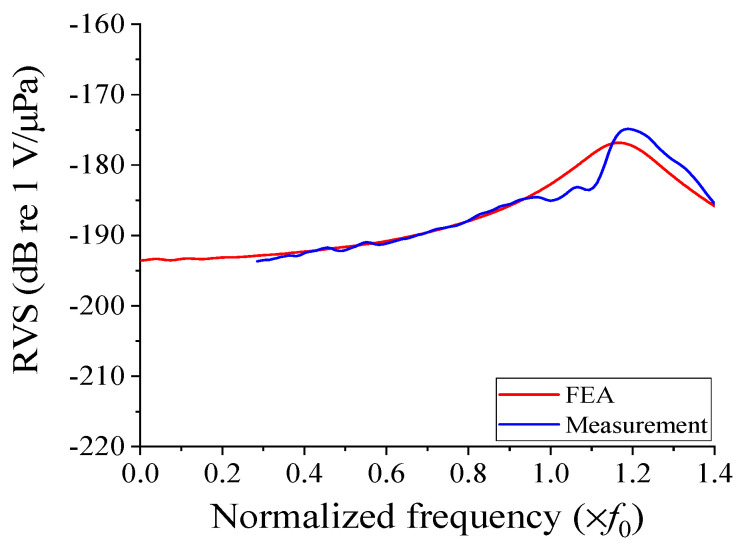
Comparison of experimental and simulated RVS spectra of the cymbal hydrophone prototype.

The RVS spectrum of the cymbal hydrophone, as measured, is presented in Figure 12 and compared to the spectrum analyzed via the FEA model. The measured spectrum encompasses the frequencies above 0.3*f*_0_ and is limited due to the size of the water tank. Over the frequency range from 0.3*f*_0_ to 0.9*f*_0_, the two sets of results exhibit an excellent agreement, with a difference of less than 0.4%. It is worth noting that the hydrophone is intended for use in the low-frequency range, making comparisons beyond 0.9*f*_0_ irrelevant. Any minor differences observed in the low-frequency range and fluctuations around the peak RVS frequency appear to be attributable to experimental errors associated with the measurement environment. This comparison affirms the validity of the optimal cymbal hydrophone structure designed in Section 4. The designed cymbal hydrophone has demonstrated higher sensitivity and a broader bandwidth compared to typical commercial hydrophones, such as spherical hydrophones, which usually have a sensitivity of about −200 dB and a fractional receiving bandwidth of up to 25% [22].

**Figure 13:**
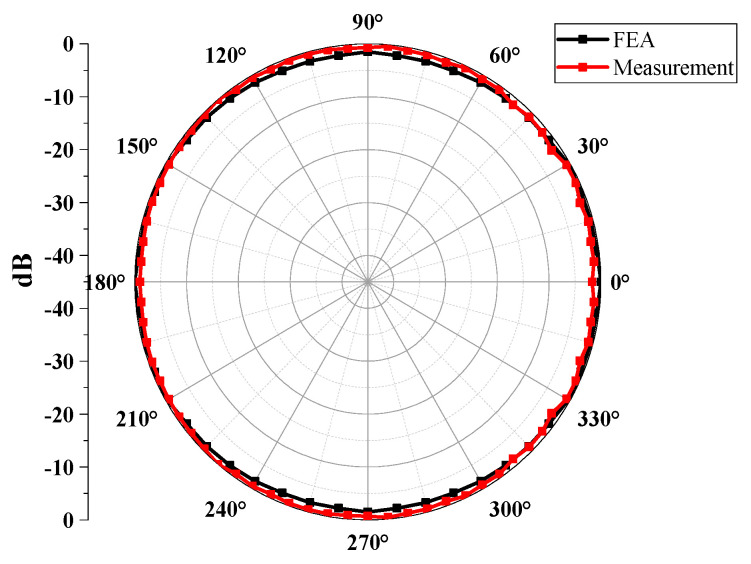
Comparison of experimental and simulated beam patterns of the prototype cymbal hydrophone.

Furthermore, we conducted measurements of the received beam pattern for the cymbal hydrophone. To assess the beam pattern, a tone burst signal centered at 0.33*f*_0_, the same frequency employed in the FEA, was applied to the projector. While the projector emitted waves, the cymbal hydrophone was rotated from 0° to 180° in 5° increments, facilitated via a computer-controlled rotator. Figure 13 provides a comparison between the measured beam pattern and the FEA-derived result. In both beam patterns, the discrepancy between the maximum and minimum gains was less than 3 dB, signifying a typical omnidirectional beam pattern. This alignment with the design specifications confirms that the cymbal hydrophone prototype exhibits the expected performance characteristics.

## 6. Conclusions

The cymbal transducer, due to its low resonant frequency relative to its size, proves to be an excellent hydrophone for applications in the low-frequency band, surpassing the capabilities of conventional hydrophones. In this study, we developed a cymbal hydrophone that would outperform existing hydrophones by offering a higher sensitivity and a wider bandwidth. For these purposes, the initial structure of the cymbal hydrophone was established, and then the effects of structural variables on the hydrophone’s performance were analyzed using the FEM. Based on the analysis results, the structure with the highest sensitivity and widest bandwidth, with a receiving voltage sensitivity level above a certain threshold, was derived using optimal design techniques. The effectiveness of our design was rigorously validated through the fabrication and testing of a cymbal hydrophone prototype, which consistently matched our design specifications.

As a result, this newly developed cymbal hydrophone boasts significantly enhanced sensitivity and a broader receiving frequency bandwidth in comparison to other hydrophones utilized for similar purposes. This advancement represents a promising leap forward in the field of underwater acoustic sensing and measurement.

## Figures and Tables

**Table 1:** Dimensions of structural parameters of the cymbal hydrophone.

Structural Parameter	Symbol	Dimension (mm)
Diameter of the cavity apex	*d*_a_	4.5
Diameter of the cavity base	*d*_b_	15.0
Diameter of the piezoceramic disk	*d*_c_	20.0
Height of the cavity	*h*_c_	0.9
Thickness of the metal cap	*t*_m_	0.5
Thickness the piezoceramic disk	*t*_c_	1.0
Width of the ring	*w*_r_	3.0
Width of the bond	*w*_b_	0.3

**Table 2:** Optimized dimensions of the cymbal hydrophone.

*d*_b_ (mm)	*h*_c_ (mm)
16.49	0.82

**Table 3:** Quantitative comparison of the performance of the initial and optimized cymbal hydrophones.

	Before Optimization	After Optimization
Peak RVS frequency	*f*_0_	1.17*f*_0_
Bandwidth	0.365*f*_0_	0.464*f*_0_
RVS level at 0.01*f*_0_	−193.30	−193.17
Bandwidth/peak RVS frequency (%)	36.54	39.77

## Data Availability

Data are contained within the article.
